# Branch Retinal Artery Occlusion in a Young Adult: A Diagnostic Clue to Systemic Embolic Risk

**DOI:** 10.7759/cureus.92291

**Published:** 2025-09-14

**Authors:** Justin Nguyen, Jasmine Q Nguyen, Julia C Moore, Briana Krutsinger, Matthew D Overturf

**Affiliations:** 1 Medical School, Edward Via College of Osteopathic Medicine, Monroe, USA; 2 Optometry, University of Houston College of Optometry, Houston, USA; 3 Ophthalmology, Edward Via College of Osteopathic Medicine, Monroe, USA; 4 Clinical Medicine, Edward Via College of Osteopathic Medicine, Monroe, USA; 5 Anatomical Sciences, Edward Via College of Osteopathic Medicine, Monroe, USA

**Keywords:** asymptomatic presentation, branch retinal artery occlusion, chronic brao, embolus, heart valve replacement, ischemic stroke, retinal ischemia

## Abstract

Branch retinal artery occlusion (BRAO) is characterized by the blockage of one or more of the smaller branches of the central retinal artery, resulting in localized retinal ischemia and potential abrupt vision impairment. Typically occurring in older adults, BRAO is frequently linked to embolic events originating from carotid or cardiac sources, such as cholesterol or calcific plaques. Conversely, BRAO in younger individuals is uncommon and often indicates an atypical systemic etiology. Cardiac conditions, including valvular disease and replacement heart valves, have been documented as embolic sources in such instances. This case report details a 41-year-old woman with a prior history of a mechanical aortic valve replacement who developed chronic BRAO potentially attributed to a suspected calcific embolus. Her evaluation revealed a calcific embolus causing inferior temporal BRAO with corresponding visual field loss and inner retinal atrophy. She was co-managed with a systemic stroke workup, cardiology, resulting in warfarin dose adjustment, and reported stable vision at six-month follow-up.

## Introduction

Branched retinal artery occlusion (BRAO) constitutes a type of retinal arterial ischemia primarily induced by embolic phenomena, with emboli generally originating from atherosclerotic plaques within the ipsilateral carotid artery or from cardiac sources such as valvular disease or atrial fibrillation [1-3]. The American Heart Association designates retinal artery occlusion (RAO) as the ocular equivalent of an acute stroke, emphasizing the necessity for prompt systemic evaluation, given that these events are frequently linked with underlying vascular risk factors and denote an elevated risk of potential subsequent cerebrovascular and cardiovascular incidents [4,5].

Retinal emboli are classified based on their composition and clinical presentation into three principal categories: cholesterol emboli, also known as Hollenhorst plaques, which predominantly originate from carotid atheromatous plaques. Fibrin-platelet emboli may originate from thrombotic or cardiac sources, whereas calcific emboli are generally associated with cardiac valvular disease [6]. Occlusion of the central retinal artery or its branches results in ischemia and subsequent atrophy of retinal tissue, which could lead to permanent vision loss [2,3]. The extent of damage is dependent on the duration and location of the occlusion. Irreversible retinal injury can commence within 90 minutes and becomes highly probable after four hours of interrupted blood flow [2]. Unlike central retinal artery occlusions (CRAOs), BRAOs tend to produce localized visual field defects and often preserve central acuity [3]. More than half of patients presenting with BRAOs retain visual acuity of greater than or equal to 20/40, with visual recovery being more common in cases of transient BRAOs compared to potential permanent situation [3].

In older adults, BRAO is predominantly associated with conventional vascular risk factors, including hypertension, diabetes mellitus, hyperlipidemia, ischemic heart disease, and tobacco use [6-8]. The incidence of RAOs is estimated at approximately 0.85 per 100,000 individuals annually, with it affecting males slightly more frequently than women, and most commonly occurring in the seventh decade of life [9]. More specifically, the incidence rate of noncentral RAO was 5.12 per 100,000 person-years, more than double that of CRAO at 2.25 per 100,000 person-years [10]. In younger patients, BRAO is relatively uncommon and warrants a comprehensive systemic investigation to assess for atypical etiologies. These may encompass congenital structural cardiac anomalies (e.g. aortic stenosis, atrial septal defects/patent foramen ovale), hypercoagulable states, systemic vasculitides, and iatrogenic causes [11,12]. Regardless of age, a diagnosis of BRAO signifies the presence of underlying systemic vascular risk factors or disease. As emphasized by the American Heart Association, a meticulous cardiovascular assessment is crucial to identifying the primary cause and mitigating the risk of subsequent events [4,5].

We present an extremely rare case of a 41-year-old woman with a pertinent surgical history of aortic valve replacement who developed chronic BRAO secondary to a calcific embolus.

## Case presentation

A 41-year-old African American female presented for a comprehensive ocular health and vision examination with a primary complaint of long-term decreased vision in the left eye (OS; derived from Latin *oculus sinister*). She reported having previously used corrective glasses but discontinued their use due to minimal perceived benefit. Her ocular history was notable for a pterygium excision in the left eye performed 11 years prior. Otherwise, no additional ocular conditions were reported. Systemically, the patient had a history of an unspecified aortic valvular disease, for which she underwent mechanical aortic valve replacement surgery eight years earlier in Israel. Details of the exact procedure are unknown and no notes were available upon request. She denied any history of hypertension, diabetes mellitus, or hyperlipidemia but had an elevated body mass index (BMI) of 35.15 kg/m². The patient’s sole reported medication was warfarin 6 mg daily, prescribed for anticoagulation therapy following her cardiac surgery. She reported monthly cardiology consultations for international normalized ratio (INR) blood testing and routine cardiac management.

**Figure 1:**
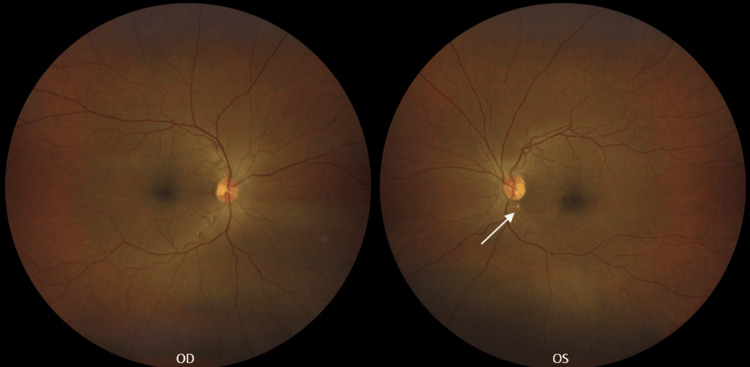
Fundus photography revealed normal findings in the right eye (OD). A branched retinal artery occlusion (BRAO) was identified in the left eye (OS), located inferior to the optic disc within the inferior temporal arcade, with a calcific embolus apparent (white arrow).

The best-corrected visual acuity (BCVA) was assessed as 20/20-2 in the right eye and 20/60 in the left eye using Snellen eye chart. Initial testing, intraocular pressures, and anterior segment examinations yielded unremarkable results in both eyes. During posterior segment evaluation of the left eye, a solitary, white, non-scintillating arterial plaque was observed at the inferior temporal arcade near the optic nerve head. This finding was also documented through fundus photography at the time of presentation and is depicted in Figure 1. Both eyes demonstrated mild hypertensive retinopathy, but the patient still denied ever having a history of high blood pressure. When inquired about possible stroke symptoms, the patient denied experiencing acute or transient vision loss, limb weakness, facial drooping, paresthesia, or dysarthria. She reported intermittent dizziness; however, it was not present at the time of examination.

**Figure 2:**
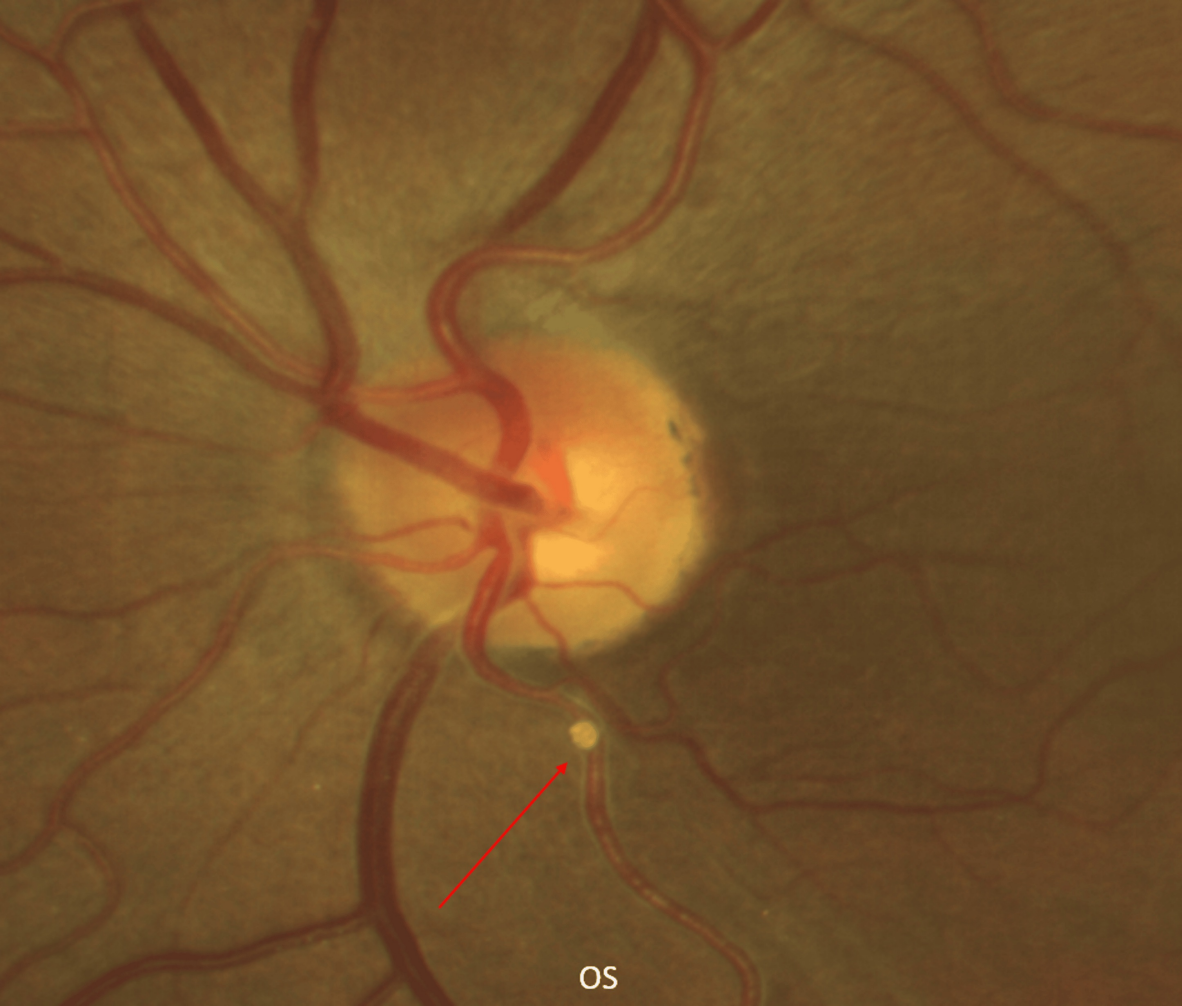
Enhanced image of calcific branched retinal artery occlusion (BRAO) in the left eye (OS). The red arrow indicates a calcific embolus obstructing the inferior temporal arcade.

A diagnosis of BRAO in the left eye was established, secondary to an arterial plaque. An enhanced image of the patient's BRAO is depicted in Figure 2. The patient was referred to her cardiologist for a prompt stroke work-up within one week, including a normal carotid ultrasound and echocardiogram. Laboratory tests revealed a subtherapeutic INR of 2.0 (therapeutic reference range: 2.0-3.0 per National Institutes of Health guidelines for mechanical heart valve anticoagulation [5,13]). Given her cardiac history, however, her cardiologist adjusted the target range to 2.5-3.0, as noted in the clinic records. Consequently, her warfarin dosage was increased from 6 mg to 7 mg daily following her cardiology assessment. The arterial plaque remained detectable during fundoscopy, and she was advised to return in six months for further retinal imaging.

**Figure 3:**
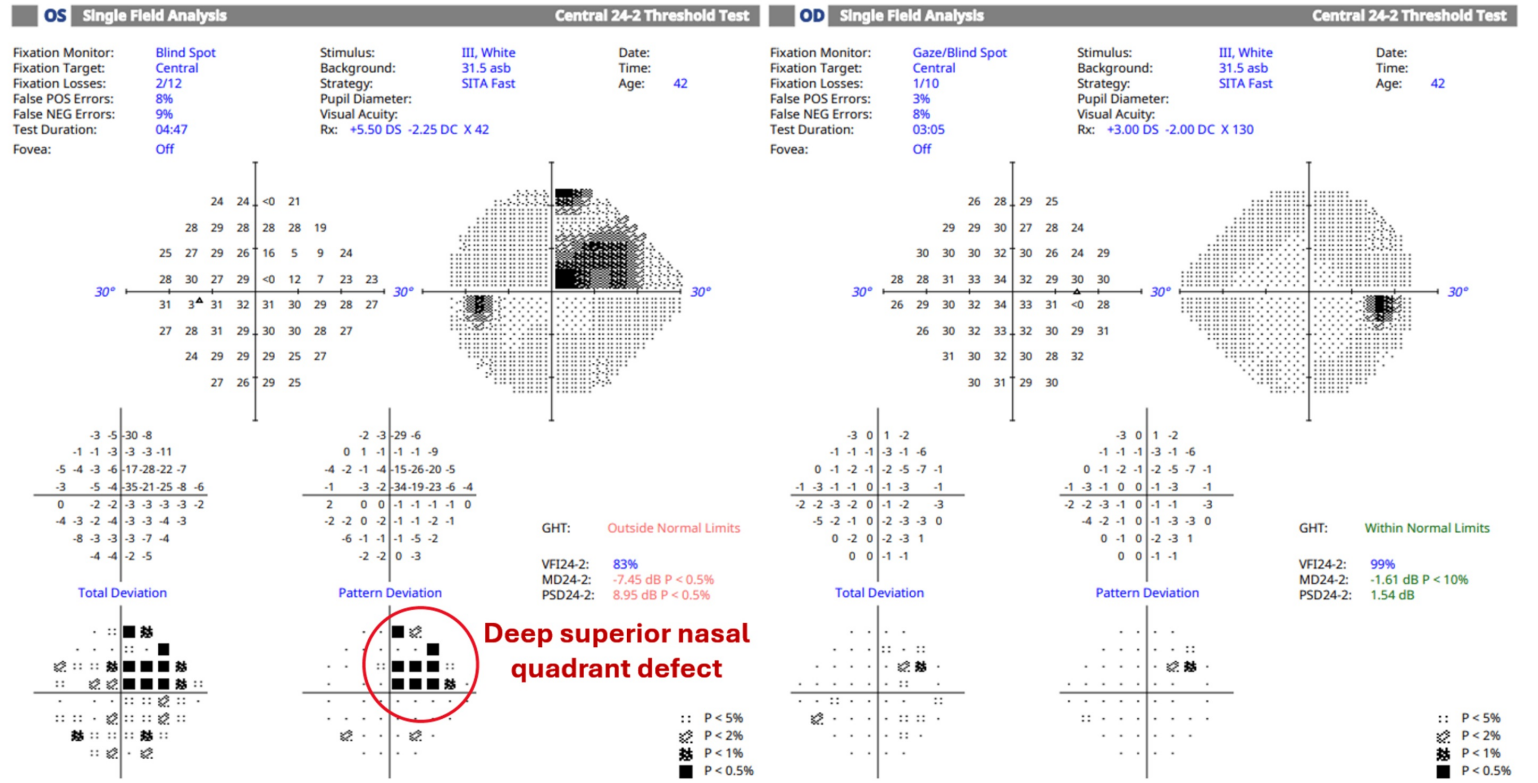
Humphrey Visual Field (24-2) results indicate a dense superior nasal quadrant defect in the left eye, consistent with ischemic damage resulting from a branch retinal artery occlusion affecting the inferior temporal retinal arcade. The left eye exhibits significantly reduced sensitivity in the affected area (VFI 83%, MD −7.45 dB, PSD 8.95 dB), with the Glaucoma Hemifield Test outside the normal limits. Conversely, the right eye maintains preserved visual function (VFI 99%, MD −1.61 dB, PSD 1.54 dB) and demonstrates a normal hemifield test. VFI - Visual Field Index; MD - Mean Deviation; PSD - Pattern Standard Deviation; OD - right eye; OS - left eye

**Figure 4:**
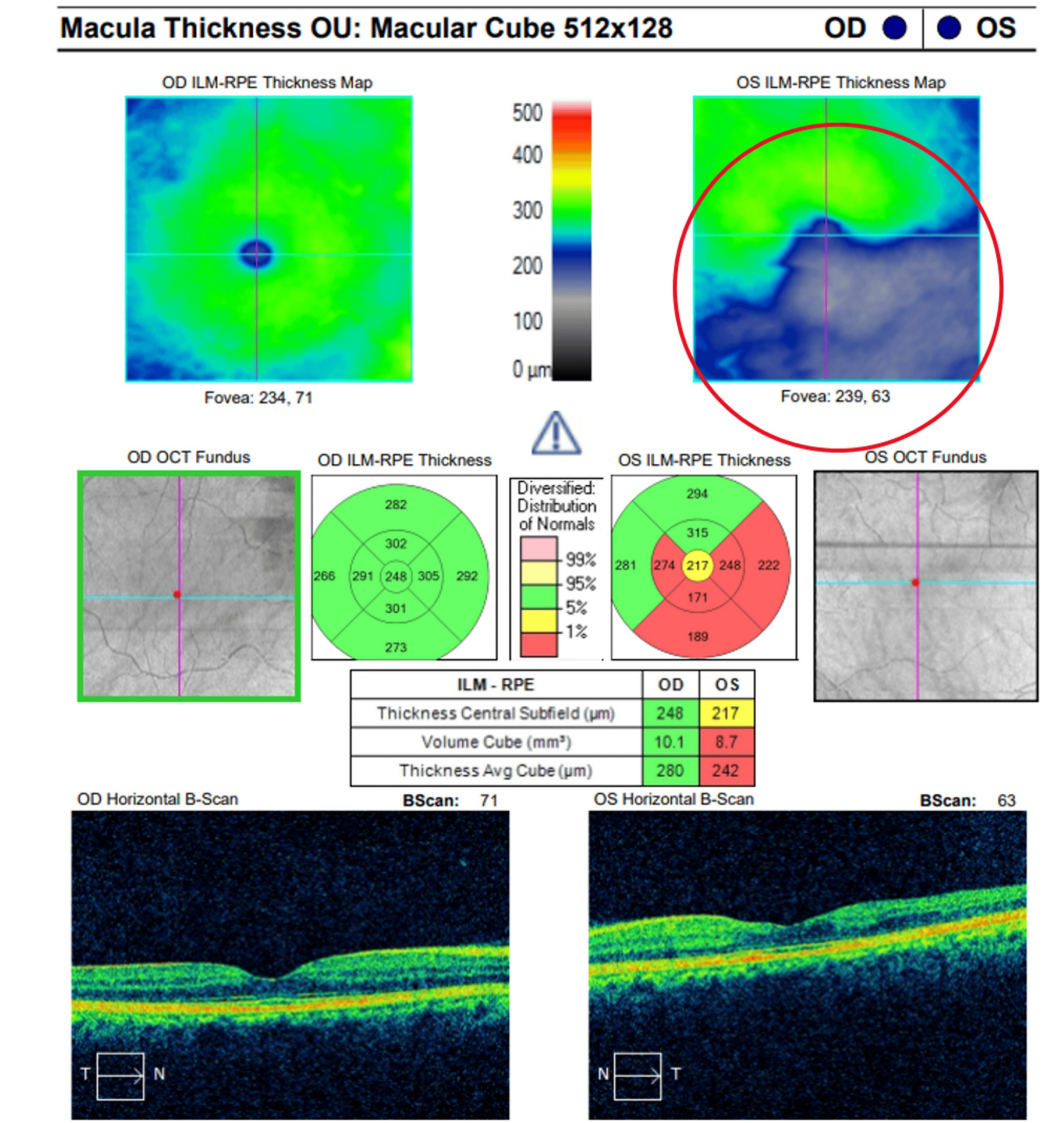
Optical Coherence Tomography (OCT) of the macula was performed on both eyes. The image of the right eye (OD) reveals normal retinal thickness and architecture. Conversely, the left eye (OS) exhibits significant thinning in the inferior macula, corresponding to a branch retinal artery occlusion (BRAO) located in the inferior temporal arcade. The area of thinning is clearly visible on the internal limiting membrane and retinal pigment epithelium (ILM-RPE) thickness map (circled) and on the cross-sectional B-scan.

**Figure 5:**
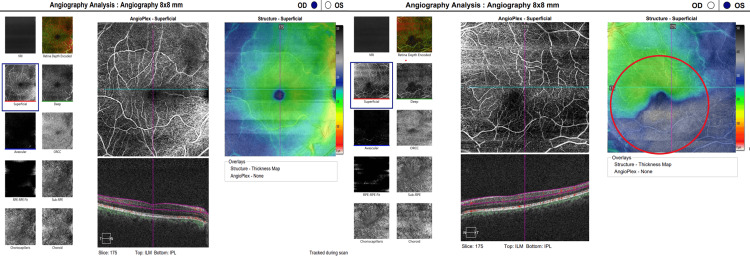
Optical coherence tomography angiography (OCT-A) of both eyes was performed. The right eye (OD) imaging demonstrates normal perfusion within the superficial capillary plexus and typical retinal architecture. The left eye (OS) imaging reveals an area of flow deficit, characterized by hypofluorescence within the superficial capillary plexus, which corresponds to the region of retinal thinning observed on the structural thickness map (red circle). These findings are indicative of ischemia resulting from branch retinal artery occlusion (BRAO) in the inferior temporal arcade.

**Figure 6:**
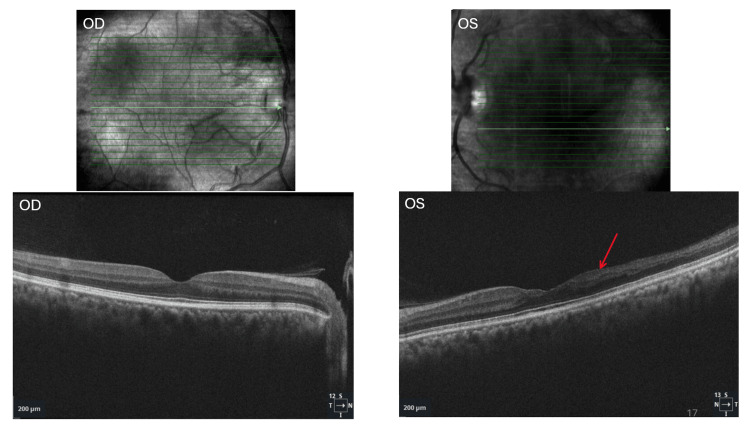
Spectral-domain optical coherence tomography (SD-OCT) of the macula demonstrating normal retinal architecture in the right eye (OD). In the left eye (OS), there is focal retinal thinning involving the inner retinal layers, including the ganglion cell layer, inner plexiform layer, inner nuclear layer, and outer plexiform layer (red arrow).

At her six-month follow-up, the patient remained asymptomatic for stroke symptoms and reported stable vision. A comprehensive evaluation was performed, including Humphrey Visual Field (HVF) 24-2 Swedish Interactive Thresholding Algorithm (SITA) Fast and Cirrus Optical Coherence Tomography (HD-OCT) angiography and macular imaging, illustrated in Figures 3-6. Strong evidence of structural and functional damage consistent with the location of the arterial blockage was displayed in imaging of the left eye. HVF revealed a dense superior nasal defect, illustrated in Figure 3, consistent with the inferior temporal retinal arcade blockage from the BRAO. This visual field defect is an expected finding from ischemic damage in the inferior temporal portion of the retina. In addition, OCT imaging, illustrated in Figures 4-6, revealed reduced inferior macular thickness with inner retinal layer atrophy and inferior capillary dropout correlating to the area of blockage. Functional visual field, macular thickness, and capillary vasculature remained preserved in the right eye.

Although the duration of the BRAO could not be conclusively determined, imaging findings indicated the presence of longstanding chronic alterations. As demonstrated in Figure 6, the presence of atrophy in the inner retinal layers is indicative of chronic ischemic damage. The outer retina, comprising the outer nuclear layer and photoreceptor layers, remains relatively preserved, underscoring the selective susceptibility of the inner retina to arterial ischemia.

A report detailing these findings was forwarded to the patient’s primary care physician and cardiologist, accompanied by recommendations for additional investigations, including hypercoagulability testing and vigilant INR monitoring. A likely and plausible etiology was a calcific embolus originating from multiple factors including her obesity status and her mechanical heart valve, particularly in the context of subtherapeutic anticoagulation and physiological turbulent flow. This clinical scenario warranted a low threshold for involving both her primary care and cardiology team to ensure a comprehensive and multidisciplinary approach to ongoing treatment. The patient also received counseling regarding the signs and symptoms of stroke and was scheduled for a four-month ophthalmology follow-up to observe her ocular and systemic health.

## Discussion

Distinguishing between the acute and chronic stages of BRAO significantly influences the urgency and strategy for systemic evaluation. Multimodal imaging has played a vital role in confirming whether this patient’s BRAO is in the acute or chronic phase. In cases of acute BRAO, early fundoscopy can yield normal findings; however, this can evolve into sectoral retinal whitening in the region supplied by the affected artery [14]. This phenomenon ultimately results in inner retinal ischemia and intracellular edema, leading to a hyperreflective, thickened, and opaque appearance of the inner retinal layers on OCT imaging [14,15]. As the acute phase progresses to resolution, retinal edema diminishes, leaving behind inner retinal atrophy indicative of irreversible ischemic damage, thereby representing the chronic phase of BRAO [2,14,15]. OCT angiography may demonstrate capillary nonperfusion within the affected area, consistent with longstanding vascular impairment [14]. The presence of both retinal atrophy and capillary nonperfusion, as evidenced in the patient’s imaging (Figures 4-6), suggests a more chronic disease trajectory.

Acute, symptomatic RAOs represent an emergent ophthalmic condition necessitating prompt systemic assessment as in this patient. Approximately 10-15% of patients with BRAO may exhibit a concurrent or recent silent cerebral infarct on neuroimaging, even in the absence of neurological symptoms [6]. The one-year risk of ischemic stroke after a BRAO is estimated at approximately 2-3%, in comparison to 3-6% following a CRAO, with the majority of strokes occurring early in the post-occlusion period [6]. Immediate referral to a stroke center for comprehensive medical evaluation is recommended; if such a facility is unavailable, an emergency room constitutes the next suitable option [3,6]. The ischemic stroke workup may include, but is not limited to, magnetic resonance angiography (MRA), carotid artery doppler ultrasound, and echocardiography [3,6]. A chronic BRAO generally does not necessitate urgent systemic evaluation; however, a thorough vascular and cardiovascular assessment remains essential if no prior embolic source has been identified [2,4].

BRAOs typically present in elderly patients with atherosclerotic risk factors such as diabetes, hypertension and/or carotid stenosis [7]. What distinguishes this case from existing literature is the potential association of BRAO with a mechanical heart valve in a relatively young patient. While CRAO is more frequently reported in this context, documented cases of BRAO remain scarce, underscoring the importance of reporting this presentation. Given this patient’s medical history, mechanical heart valve replacement is a recognized risk factor for systemic and cerebral embolic events resulting from altered hemodynamics [11,16]. Turbulent shear forces generated by mechanical and prosthetic valves can cause fragmentation of red blood cells, leading to schistocyte formation, hemolytic anemia, and a prothrombotic state [9,11]. Abnormal flow patterns may elevate endothelial stress, thereby facilitating the dislodgment of cholesterol and calcific debris from vascular plaques [9,11]. Although the association between mechanical valves and BRAO is scarcely documented, the underlying pathophysiology lends plausibility to this relationship and makes this an interesting, but challenging case [8,16,17]. Most reported calcific embolic retinal events in this context involve CRAO, which is likely attributable to the larger caliber and more direct course of the central artery [8,15,18]. In our patient, the presence of a mechanical valve provides a plausible increased risk of embolus. Although mechanical valves have been linked more often with CRAO, the possibility of BRAO in this context should be considered, particularly when no alternative embolic source is identified per her normal echocardiogram and bilateral carotid doppler ultrasounds. BRAO attributable to a mechanical heart valve may be underdiagnosed, especially in younger or asymptomatic patients, due to subtler clinical presentations, transient episodes, or unrecognized embolic sources [8,18].

In this instance, subtherapeutic anticoagulation following mechanical valve replacement may also have contributed to the development of the BRAO via mechanisms such as altered blood viscosity and flow [11,13]. Although the patient reported regular follow-up with cardiology and routine INR monitoring, her INR level was below the therapeutic range at the time of evaluation. Despite her stated adherence to the anticoagulation regimen, enhanced patient education regarding the importance of consistent daily dosing, potential drug and dietary interactions, and the signs of subtherapeutic anticoagulation could have potentially lowered the risk of developing an embolic complication. Additional preventive measures might have included more frequent INR testing during periods of fluctuation or home INR self-monitoring to maintain consistent therapeutic levels. Moreover, routine ophthalmic screening and patient education concerning the specific warning signs of retinal artery occlusion may have enabled earlier detection and intervention.

The patient’s elevated body mass index (BMI) may have further increased thrombotic risk, underscoring the importance of comprehensive cardiovascular risk factor modification, including weight reduction, lipid management, and blood pressure control. Elevated BMI is independently associated with a higher risk of thromboembolic events, such as deep vein thrombosis and pulmonary embolism, in a dose-response manner, with abdominal adiposity serving as an even stronger predictor of risk [19,20]. Excess adiposity promotes a prothrombotic state through endothelial dysfunction, increased platelet activation, elevated levels of von Willebrand factor, tissue factor, clotting factors VII and VIII, fibrinogen, and impaired fibrinolysis, changes driven by chronic low-grade inflammation and oxidative stress [21]. These abnormalities have been demonstrated to improve with weight loss. Since much of the cardiovascular and cerebrovascular risk associated with elevated BMI is mediated through hypertension, dyslipidemia, and hyperglycemia, achieving a BMI within the recommended range and managing these factors may help reduce thrombotic risk across both arterial and venous systems, indicating that the patient’s excess weight could have contributed to her BRAO [22].

Our case directly addresses this issue, finding that while BRAOs have less risk for stroke than CRAOs, the risk is still elevated to warrant referral to specialized care. Although BRAO carries a lower risk of subsequent ischemic stroke than CRAO, its occurrence should prompt urgent systemic evaluation and aggressive risk-factor modification to reduce the likelihood of cerebrovascular events. The occurrence of RAO in this case of a young adult should raise a high index of suspicion for uncommon embolic sources, including structural cardiac abnormalities and complications related to prosthetic/mechanical valves. Although most reported cases of prosthetic valve-related emboli involve CRAO, anecdotal evidence and limited case reports suggest that BRAO may also occur in this setting [23]. Further research is warranted to better define this association and guide screening. This case underscores the need for improved coordination between ophthalmology, cardiology, and primary care to optimize preventive care and reduce the risk of embolic ocular events in patients with a heart valve replacement.

## Conclusions

Considering the long-term repercussions of embolic incidents in patients with mechanical valves, it is imperative to establish coordinated care among ophthalmology, cardiology, and primary care professionals. Maintaining therapeutic INR levels through regular anticoagulation monitoring may reduce the likelihood of subsequent vascular events. Furthermore, conducting routine retinal examinations in high-risk populations (e.g. reverse shunting heart diseases, diabetes, hypertension) can enable earlier identification of embolic phenomena and permit intervention prior to the development of irreversible visual impairments.

This case highlights the importance of heightened awareness regarding BRAO as a potential presentation of systemic embolic disease, particularly in younger individuals with mechanical heart valves. Younger patients with such prostheses should receive counseling concerning the potential risk of RAO, which, although infrequent, constitutes a serious embolic complication of aortic valve replacement. Patient education is vital to ensure they are cognizant of warning signs such as sudden, painless vision loss or visual field defects, and the necessity of seeking immediate ophthalmologic assessment should these symptoms manifest. We believe this represents an extremely rare case of calcific branch retinal artery occlusion in a young patient. Further investigation into the prevalence and underlying mechanisms of retinal artery occlusion within this demographic may contribute to the development of enhanced screening protocols, anticoagulation management, and interdisciplinary follow-up strategies, thereby optimizing ocular and systemic health outcomes.
